# Copaiba More Active When Combined with Purgatives

**Published:** 1845-04

**Authors:** 


*Copaiba more active when combined with Purgatives.* By M. Jacquetant.—M. Jacquetant gives a full account of the hospital practice of M. Diday in the cure of gonorrhœa, by which it appears that the balsam of copaiba acts much more energetically in the removal of that disease if combined with purgatives. Many instructive cases are shortly given of this mode of treatment, and compared with others in which the copaiba was administered alone, and also with others in which the cure was attempted by means of purgatives alone. The formula which M. Diday found most successful was three drachms of the balsam of copaiba, 4½ drachms of the powder of cubebs, and 45 grains of the powder of jalap made into an electuary, of which the half is taken in the morning, and the remainder in the evening. The treatment rarely lasted more than five days, by which time a permanent cure was generally effected.—*Ibid, from Gaz. Med. de Paris.*

